# ICF and perception of functioning according to children/adolescents in follow-up with speech/language disorders

**DOI:** 10.1590/2317-1782/20232021167

**Published:** 2023-08-11

**Authors:** Amanda Brait Zerbeto, Maria de Lurdes Zanolli, Regina Yu Shon

**Affiliations:** 1 Pós-graduação em Saúde da Criança e do Adolescente, Universidade Estadual de Campinas - UNICAMP - Campinas (SP), Brasil.

**Keywords:** International Classification of Functioning, Disability and Health, Language Disorders, Patient Participation, Communication Barriers, Speech Language and Hearing Sciences, Clinical Evolution, Classificação Internacional de Funcionalidade, Incapacidade e Saúde, Transtornos da Linguagem, Participação do Paciente, Barreiras de Comunicação, Fonoaudiologia, Evolução Clínica

## Abstract

**Purpose:**

To characterize changes in the functioning aspects, in the perception of children and adolescents with speech and language disorders under speech-language follow-up, using the ICF.

**Methods:**

Descriptive, analytical and longitudinal research, with a qualitative and quantitative approach, whose sample consisted of 60 children and adolescents: 30 with speech and language disorders and 30 with typical speech and language development. Data collection was carried out in two moments: beginning of the research and six months later. A semi-structured questionnaire was administered to the participants, and a medical records analysis was performed. From these data, functioning was classified using he ICF categories. The Wilcoxon test and thematic content analysis were used to compare the interviews.

**Results:**

The use of ICF allowed characterizing changes resulting from speech-language follow-up. Participants with speech and language disorders presented a decrease in the magnitude of the qualifiers in the categories: articulation and fluency, social relationships, daily activities, engagement in play, people's attitude barriers, and how to handle stress.

**Conclusion:**

The findings show changes in components of Body Functions, Activities and Participation, and the influence of Environmental Factors after speech-language follow-up, in the perception of the studied group, which brings relevant subsidies for a greater understanding of functioning and therapeutic intervention. The use of the ICF enabled the longitudinal analysis in a biopsychosocial approach, contemplating, in addition to biological aspects, the social impact of speech and language disorders in the lives of these children and adolescents.

## RESUMO

**Objetivo:**

Caracterizar mudanças nos aspectos de funcionalidade, segundo a percepção de crianças e adolescentes com alterações de fala e linguagem em acompanhamento fonoaudiológico, utilizando a Classificação Internacional de Funcionalidade, Incapacidade e Saúde (CIF).

**Método:**

Pesquisa de delineamento descritivo-analítico, longitudinal e de abordagem qualitativa-quantitativa, cuja amostra se constituiu de 60 crianças e adolescentes: 30 com alteração de fala e linguagem e 30 com desenvolvimento típico de fala. Coleta foi realizada em dois momentos: início da pesquisa e seis meses depois. Foi realizada entrevista com questionário semiestruturado com os participantes, e análise de prontuário. A partir desses dados, a funcionalidade foi caracterizada com as categorias da CIF. Para comparação entre as entrevistas utilizou-se o Teste Wilcoxon e análise de conteúdo temática.

**Resultados:**

O uso da CIF possibilitou caracterizar mudanças ocorridas com o acompanhamento fonoaudiológico. Os participantes com alterações de fala e linguagem apresentaram diminuição na magnitude dos qualificadores nas categorias de funções de articulação e fluência, relacionamentos sociais, atividades do cotidiano, envolvimento no brincar, atitudes barreiras das pessoas, modo de lidar com o estresse.

**Conclusão:**

Os achados mostram mudanças nos componentes de Funções do Corpo, Atividades e Participação e a influência dos Fatores Ambientais após o acompanhamento fonoaudiológico na percepção do grupo estudado, o que traz subsídios relevantes quanto à funcionalidade para maior compreensão e intervenção terapêutica. A utilização da CIF possibilitou estudo e análise longitudinal em uma abordagem biopsicossocial, contemplando além dos aspectos biológicos, o impacto social das alterações de fala e linguagem na vida dessas crianças e adolescentes.

## INTRODUCTION

The perception of children and adolescents with speech and language disorders on the changes that occur in their speech and daily life after speech-language follow-up has been little discussed in the literature. Knowing their perception can provide important data on functioning and influence of environmental factors for therapy. The International Classification of Functioning, Disability and Health (ICF)^(1)^ is a framework to be considered by speech-language pathologists (SLP) for this purpose. The ICF allows characterizing aspects of functioning throughout the therapeutic process and from the perspective of different people involved, like children, parents, and health professionals.

ICF, a World Health Organization (WHO) classification, interests those working in the field of language by promoting a change from the biomedical view of health to a biopsychosocial view of the person^(1-9)^. The ICF system is divided into components: Body Structure, Body Functions, Activities and Participation, and Environmental Factors. Each component is composed of domains and categories. Its relevance is especially due to the fact that it addresses functionality and disability as a dynamic interaction between health states and contextual factors^(1)^. Functioning indicates the positive aspects of interaction between an individual (health condition, body components, activity and participation) and their contextual factors. Disability indicates the negative aspects of this interaction.

The biopsychosocial perspective of ICF^^1^^ considers important categories to be observed in speech-language follow-up, such as the characterization of speech and language difficulties and their impact on social relations and everyday activities^(4-5,10-13)^.

Another possibility offered by the ICF is the qualitative (describing aspects of impairment, difficulties and facilitators/barriers) and quantitative (using the qualifiers) category analysis. By using these two types of analysis, it is possible to describe and characterize the magnitude of functioning, which are important concepts of the ICF.

Since the ICF presents a standardized language, it can be used in every process of therapy, such as initial characterization^(5,14-15)^, development of treatment goals^(4,14,16-17)^ and evaluation of therapy outcomes^(12,14,18)^. The use of ICF throughout the therapeutic process is interesting because it ensures that the SLP will observe the changes that occurred after speech-language follow-up in the different components.

ICF provides a unified language to describe health conditions, and allows elaboration of instruments to characterize functioning through different points of view. Analysis of the effects of the speech-language follow-up has been carried out mainly under the perspective of SLPs and parents of children and adolescents with speech and language disorders^(12,14,18-21)^. Examples, where speech-language pathologists and parents evaluated these changes have taken place in Canada using the Focus on the Outcomes of Communication Under Six (FOCUS)^(12,15,18)^. In these studies, parents reported more positive changes in Activities and Participation and Personal Factors than clinicians^(12)^. In this way, the importance of listening to the family’s perception and involving them throughout the therapeutic process is recognized^(6,10,17,22)^.

One tool that addresses children’s perception is the Speech Participation and Activity of Children (SPAA-C)^(4)^. The SPAA-C is based on the ICF and contains questions to be answered by the child, siblings, friends, parents and teachers. Listening to children about how they deal with their difficulties is important because speech and language disorders can have a different impact on each child's life. There is evidence that only the individuals with speech and language disorders are able to report, as they are the ones who are dealing with their own daily difficulties^(4)^.

Because the ICF contains relevant aspects to SLPs who work with speech and language disorders, it is important to study their use in the therapeutic process. Researches have addressed parents’ and professionals’ perception of speech disorders^(12,14,18-21)^, but few studies have investigated children’s and adolescents’ perception of their own difficulties. Therefore, the aim of this study was to characterize changes in functioning aspects as perceived by children and adolescents with speech and language disorders, under speech-language follow-up, using the ICF.

## METHODS

This is a descriptive-analytical research with a longitudinal design and a qualitative-quantitative approach. Two groups of 30 children and adolescents, aged between four and 16 years, participated in the study. It was excluded individuals who presented hearing loss or neurological problems, who had difficulty understanding the interview questions and those who could not answer orally to the questions. Data collection on changes in language, participation and functioning aspects was performed in two moments, with a six to eight-month interval. The Ethics Committee approved the research by the CAAE 14110313.9.0000.5404.

The group of children and adolescents with speech and language disorders (SLD) was composed of 30 participants in a speech-language pathology school clinic. All children and adolescents with speech and language disorders in attendance at the clinic were included. The main diagnostic hypothesis in the SLD group was stuttering (15 cases) and language disorders (15 cases). Some participants presented both speech and language disorders (five)^(23)^, in these cases it was considered the main diagnostic. In the institution, language evaluations are usually performed with observational methods and a naturalistic situation, with little use of standardized tests. Evaluations in naturalistic situations are not structured (or are little structured), the proposal foresees the observation of the child in a situation that is characterized as the most natural as possible. It seeks to observe social interaction, communicative exchanges, discursive initiative and spontaneity^(24)^.

SLP sessions were held weekly, each lasting one hour. As part of the treatment to stutterers, a therapeutic group with their relatives happened monthly. The purpose of the group was for the family members to exchange experiences and talk to health professionals. In cases of speech disorders, as the participants participated in different internships and supervisors, groups with family members were not always possible.

The group with typical speech and language development (TD) was composed of 30 participants, selected by drawing lots, from two public schools in a Brazilian city in the interior of the State of São Paulo, Campinas. The intention of presenting the results of both groups in interviews 1 and 2 was to use a “reference standard”, because at the time of designing the study there were no publications with results in typical children.

To carry out the research the following steps were taken: i) literature review surveying papers on ICF and speech and language disorders; ii) reading all ICF chapters related to speech and language; iii) pre-selection of second-level ICF categories; iv) development of questions guiding the interview from the SPAA-C^(16)^ and other studies, and the pre-selected ICF categories; v) conducting interviews; vi) transcript of interviews; vii) review of pre-selected ICF categories (all categories of step iii were selected); viii) re-reading of transcribed interviews, analysis of video and audio data, and analysis of medical records for the final qualification of each participant’s categories.

For data collection, individual interviews were conducted with participants using a semi-structured questionnaire (questions about people who they like and don’t like to talk to, what people say about their language/speech, how they feel when talking with different people) and medical record analysis. Semi-structured questions guiding the interview were applied by the SLP researcher who conducted the interview^(5)^. Interviews were videotaped in the SLD group and recorded in audio in the TD group. Interviews were transcribed for data analysis. Closed questions were applied to parents for collecting socio-demographic data, presented in Table 1.

**Table 1:** Characterization of participants of Groups with speech and language disorders (SLD) and with typical speech and language development (TD)

Variable		SLD N(%)	TD N(%)
Gender	Female	6 (20)	16 (53.3)
	Male	24 (80)	14 (46.7)
Age group^(25)^	Child (“9 years)	20 (66.7)	25 (83.3)
	Adolescent (≥10 years)	10 (33.3)	5 (16.7)
Per capita Income*	No information	0 (0)	7 (23.3)
	≤ 1 MW	21 (70)	12 (40)
	>1 MW	9 (30)	11 (36.7)
Mother’s Education	≤ 9 years of school	11 (36.7)	5 (16.7)
	10-12 years of school	12 (40)	17 (56.7)
	> 12 years of school	7 (23.3)	8 (26.7)
Mother’s Profession	No income (homemaker)	4 (13.3)	2 (6.7)
	With income (working with or without employment relationship )	26 (86.7)	28 (93.3)
Parents’ Marital Status	Living together	26 (86.7)	20 (66.7)
	Separated	4 (13.3)	10 (33.3)
History of language problems in family	With history	11 (36.7)	8 (26.7)
	Without history	19 (63.3)	22 (73.3)

* 1 Minimum wage (MW) in Brazil in 2015 = R$ 788.00 (= about US$263 considering US$1 equals R$3)

**Caption:** N (number of cases); % (percentage)

For the definition of category qualifiers, we considered: participants' answers; observations made in the analysis of interview videos; and medical records information in the SLD group. For the qualification of ICF categories, we followed the instructions recommended by the WHO^(1)^ on the definitions, inclusions and exclusions of each category. The qualifiers range from zero (0), corresponding to no difficulty or impairment, to one (1), two (2), three (3), or four (4), which lists a complete difficulty or impairment. In Environmental Factors, qualifiers can be facilitators or barriers. Three judges with experience in ICF agreed on the qualifiers used, each made his classification and was considered the qualifier in which there was agreement between at least two judges.

The qualification of ICF categories was performed individually for each participant, considering all information collected. Information regarding the Body Functions of the SLD Group was qualified mainly with data obtained from reports and examinations of the records, evaluations in naturalistic situations, and interview data. Data about the Activity and Participation categories were collected from the interviews and video and audio analysis, which provided data on the participants' perception of their own difficulties. For the classification of the categories of Environmental Factors, data from the interviews and information of speech-language reports contained in the medical records were used, in the case of the SLD group.

To exemplify the selection of qualifiers, we report the category d750 (Informal social relationships). Question 11, asked "How do you feel when you talk to your friends? Why?". Participants who selected the smiley face were classified with the qualifier "0". If they selected the neutral face, they had the qualifier 1 or 2; the choice of the sad face implied the selection of qualifiers 2, 3 or 4. The selection of qualifiers was carried out according to the reasons and explanations given for the choice of this expression.

Data of the present research were analyzed with a quantitative and qualitative approach, because we considered it important to use qualifiers and to describe the functioning and disability. The quantitative analysis was performed using the software for statistical analysis SPSS for Windows (version 16.0). The Wilcoxon test was used to compare the changes in the ICF qualifiers of the same group between the first and second interviews. The significance level adopted for the statistical tests was 5% (p≤0.05).

In the qualitative analysis, the transcribed interviews were read, re-read and submitted to thematic content analysis^(26)^.

## RESULTS

In Body Functions, the SLD group presented statistically significant changes from the first to the second interview in the b320 (articulation) and b330 (fluency and rhythm) categories (Table 2). In the articulation functions category there was an increase from 12 to 20 participants with 0 (no impairment), and in the Fluency and Rhythm Functions category there was an increase from 14 to 18 participants with no difficulty. Of the 30 participants in the SLD group, 10 were discharged from speech-language follow-up right before the second interview. Most of the 20 participants who remained in attendance noticed improvement in their speech. Participants reported that the improvement was also perceived by people who live with them, and that the instances in which they were not understood had decreased.

**Table 2:** ICF classification of Body Functions in the Groups with speech and language disorders (SLD) and with typical speech and language development (TD) in Interviews 1(I1) and 2(I2)

Categories/ Qualifier*	Group		.0 n(%)	.1 n(%)	.2 n(%)	.3 n(%)	.4 n(%)	p-value**
b117- Intellectual functions	SLD	I1	30(100)					1.00
		I2	30(100)					
	TD	I1	30(100)					1.00
		I2	30(100)					
b167-Mental functions of language	SLD	I1	30(100)					1.00
		I2	30(100)					
	TD	I1	30(100)					1.00
		I2	30(100)					
b230- Hearing functions	SLD	I1	30(100)					1.00
		I2	30(100)					
	TD	I1	30(100)					1.00
		I2	30(100)					
b320- Articulation functions	SLD	I1	12(40)	9 (30)	4(13.3)	5(16.7)		<0.01
		I2	20(66.7)	6(20)	2(6.7)	2(6.7)		
	TD	I1	30(100)					1.00
		I2	30(100)					
b330- Fluency and rhythm of speech functions	SLD	I1	14(46.7)	6(20)	8(26.7)	2(6.7)		<0.01
		I2	18 (60)	8(26.7)	3(10)	1(3.3)		
	TD	I1	30(100)					1.00
		I2	30(100)					

* 0 (no impairment), 1 (mild impairment), 2 (moderate impairment), 3 (severe impairment), 4 (complete impairment);

** Wilcoxon Test (p≤0.05). I1 (Interview 1), I2 (Interview 2)

Participants with moderate and severe impairment in the b320 and b330 categories continued to report difficulty in being comprehended by others. Despite this difficulty, in interview 2 it was easier to understand their speech and the participants used fewer gestures to communicate, which can improve the performance of activities and the participation.

In Table 3, nine of the ten Activity and Participation categories presented a statistically significant decrease in the degree of difficulty in the SLD group, different result from TD group (Table 4). Activities such as speaking (d330), conversing (d350), interacting (d710), relating with various partners (d730, d750, d760), and engaging in play (d880) became less difficult for children and adolescents with speech and language disorders.

**Table 3:** ICF classification of Implications in Activities and Participation in Group with speech and language disorders (SLD) in interviews 1(I1) and 2(I2)

Categories/Qualifier*		.0 n(%)	.1 n(%)	.2 n(%)	.3 n(%)	.4 n(%)	p-value**
d230- Carrying out daily routine	I1	13(43.3)	8(26.7)	8(26.7)	1(3.3)		<0.01
	I2	21(70)	3(10)	5(16.7)	1(3.3)		
d240- Handling stress and other psychological demands	I1	2(6.7)	15(50)	11(36.7)	2(6.7)		<0.01
	I2	14(46.7)	8(26.7)	6(20)	2(6.7)		
d3101- Comprehending simple spoken messages	I1	30(100)					1.00
	I2	30(100)					
d330- Speaking	I1		11(36.7)	15(50)	4(13.3)		<0.01
	I2	12(40)	12(40)	3(10)	3(10)		
d350- Conversation	I2	5(16.7)	11(36.7)	10(33.3)	4(13.3)		<0.01
	I2	20(66.7)	5(16.7)	3(10)	2(6.7)		
d710- Basic interpersonal interactions	I1	4(13.3)	15(50)	8(26.7)	3(10)		<0.01
	I2	17(56.7)	7(23.3)	5(16.7)	1(3.3)		
d730- Relating with strangers	I1	1(3.3)	10(36.7)	17(56.7)	2(6.7)		<0.01
	I2	8(26.7)	13(43.3)	9(30)			
d750- Informal social relationships (friends, colleagues)	I1	3(10)	16(53.3.)	8(26.7)	3(10)		<0.01
	I2	17(56.7)	7(23.3)	3(10)	3(10)		
d760- Family relationships (parents, siblings)	I1	5(16.7)	11(36.7)	10(33.3)	4(13.3)		<0.01
	I2	17(56.7)	5(16.7)	7(23.3)	1(3.3)		
d920- Recreation and leisure	I1	16(53.3) 9(53.3)	10(33.3)	3(10)	1(3.3)		<0.05
	I2	23(76.7)	5(16.7)	1(3.3)	1(3.3)		

* 0 (no difficulty), 1 (mild difficulty), 2 (moderate difficulty), 3 (severe difficulty), 4 (complete difficulty);

** Wilcoxon Test (p≤0.05). I1 (Interview 1), I2 (Interview 2)

**Table 4:** ICF classification of Implications in Activities and Participation in Group with typical speech and language development (TD) in Interviews 1(I1) and 2(I2)

Categories/ Qualifier*		.0 n(%)	.1 n(%)	.2 n(%)	.3 n(%)		.4 n(%)	p-value**	
d230- Carrying out daily routine	I1	30(100)						1.00	
	I2	30(100)							
d240- Handling stress and other psychological demands	I1	30(100)						1.00	
	I2	30(100)							
d3101- Comprehending simple spoken messages	I1	30(100)						1.00	
	I2	30(100)							
d330- Speaking	I1	30(100)						1.00	
	I2	30(100)							
d350- Conversation	I1	30(100)						1.00	
	I2	30(100)							
d710- Basic interpersonal interactions	I1	24(80)	6(20)					0.25	
	I2	27(90)	3(10)						
d730- Relating with strangers	I1	4(13.3)	13(43.3)	13(43.3)				0.46	
	I2	5(16.7)	14(46.7)	11(36.7)				
d750- Informal social relationships (friends, colleagues)	I1	27(90)	3(10)					0.56	
	I2	28(93.3)	2(6.7)						
d760- Family relationships (parents, siblings)	I1	24(80)	6(20)					0.01	
	I2	29(96.7)	1(3.3)						
d920- Recreation and leisure	I1	27(90)	3(10)					0.15	
	I2	29(96.7)	1(3.3)						

* 0 (no difficulty), 1 (mild difficulty), 2 (moderate difficulty), 3 (severe difficulty), 4 (complete difficulty);

** Wilcoxon Test (p≤0.05). I1 (Interview 1), I2 (Interview 2)

The SLD participants presented a great positive change in the speaking category (d330), in the question in which they marked their feelings about their speech. The sad feeling about this category was marked by any of the participants in Interview 2, unlike Interview 1. In Interview 2, participants reported being happier of how they express themselves because the speech is better, or because people comment less negatively about this aspect.

Participants of the SLD group also had less difficulty in conversing (d350). In interview 2, they said they felt happier talking to their parents (d760), followed by friends (d750) and then brothers (d760). Speech and language could still be altered, but they reported liking to talk more often than in interview 1. In interview 2 they reported a preference for talking to family members, but some participants reported they prefer to talk to friends, a situation little mentioned in interview 1. Participants like to talk to these people because they understand their speech, are fun and they have common interests.

Most of the participants in the SLD group did not report difficulties in performing daily routine tasks (d230) in interview 2. Difficulties in the school routine (such as presentation of work and getting their doubts cleared) were reported less frequently in the second interview. However, some difficulties show that certain daily routine activities are still impaired in this group: how to order a snack in the school cafeteria; and send an e-mail to clear doubts in math.

In relation to handling stress (d240), the difficulties of the SLD participants still persisted in interview 2, but they reported better coping with the situation. They less often gave up speaking when people failed to comprehend them and told people not to interrupt them while they were talking. However, for many of them, it is still not easy to deal with the situation, avoiding contact with people who call them nicknames or say something negative about their speech.

Participants said they did not like to talk with colleagues who called them nicknames, imitated the way they spoke or disturbed them when they tried to speak. Some participants reported situations of imitations or nicknames for teachers, a situation that was cited more often in interview 2. Other participants simply ran away from the situation and reported feeling sad when this happened. Participant 23 from the SLD group reported:

"*I ignore it first, until it happens again, it’s a right and wrong attitude at the same time, like I do not retort as they say, but sometimes I talk about them a little bit. Like a stutterer. It’s not meant to be ugly, it’s not to hurt, but for them to feel the knife they stick in me stuck in them.*"

In Environmental Factors, participants in the SLD and TD groups presented statistically significant changes between the first and second interviews in the categories related to individual attitudes of immediate family members (e410) and friends (e420) (Table 5). Attitudes became more facilitative.

**Table 5:** ICF classification of Environmental Factors in the Groups with speech and language disorders (SLD) and with typical speech and language development (TD) in Interviews 1(I1) and 2(I2)

Categories/ Qualifier*	Groups		.4	.3	.2	.1	+0	+1	+2	+3	+4	p-value**
e410-Individual attitudes of immediate family members (parents, siblings)	SLD	I1 (n)		4	13	7	2	3	1			<0.01
		%		13.3	43.3	23.3	6.7	10	3.3			
		I2 (n)		2	2	6	6	8	3	3		
		%		6.7	6.7	20	20	26.7	10	10		
	TD	I1 (n)					3	2	8	10	7	0.01
		%					10	6.7	26.7	33.3	23.3	
		I2 (n)					1	1	5	12	11	
		%					3.3	3.3	16.7	40	36.7	
e420- Individual attitudes of friends	SLD	I1 (n)		1	6	10	9	3	1			<0.01
		%		3.3	20	33.3	30	10	3.3			
		I2 (n)			1	7	7	5	6	4		
		%			3.3	23.3	23.3	16.7	20	13.3		
	TD	I1 (n)				2		1	7	13	7	<0.01
		%				6.7		3.3	23.3	43.3	23.3	
		I2 (n)					2		2	7	19	
		%					6.7		6.7	23.3	63.3	
e425- Individual attitudes of acquaintances, peers, colleagues, and community members	SLD	I1 (n)		4	4	16	6					<0.01
		%		13.3	13.3	53.3	20					
		I2 (n)		1	5	3	19	1	1			
		%		3.3	16.7	10	63.3	3.3	3.3			
	TD	I1 (n)			1		29					0.10
		%			3.3		96.7					
		I2 (n)					28	2				
		%					93.3	6.7				

* .4 (complete barrier), .3(severe barrier), .2 (moderate barrier), .1(mild barrier), 0 (no facilitator), +1 (mild facilitator), +2 (moderate facilitator), +3 (severe facilitator), +4 (complete faciltator);

** Wilcoxon Test (p≤0.05). I1 (interview 1), I2 (interview 2)

Participants of the SLD group reported in the second interview that close family members continued with some barrier attitudes, such as interrupting or correcting their speech, and giving instructions of what to do to speak better. However, reported attitudes occurred less often and with fewer participants. Most of these changes were reported by participants diagnosed with stuttering.

Other facilitating attitudes of family members (e410) were also reported, such as waiting for the participant to speak or not to complete their speech. One of the facilitating attitudes was reported by the mother of participant 16-SLD, who made copies of a leaflet she received at the SLP service. The booklet explained the concept of stuttering and how to deal with it, and the mother handed it over to their son’s teachers. After this attitude, she noticed an improvement in the student-teacher relationship. Her son confirmed in the interview that the relationship with his teachers and colleagues improved.

Participants of the SLD group cited more facilitative attitudes from their friends (e420) in interview 2. As an example, friends commented that their speech had improved and praised their performance in some activities. The attitudes of school mates (e425) continued to be a barrier or a neutral factor for participation, but were described as less barrier than in the first interview.

When asked how they felt when speaking with their SLP, most participants with speech and language disorders said they felt happy in interviews 1 and 2. Some of the reasons they felt this way were: they did not feel they were being judged for speaking right or wrong; they felt more relaxed and stuttered less speaking with the professionals; and because SLP were there to help them improve communication.

## DISCUSSION

The use of the ICF allowed the SLP to characterize the changes of therapy through a biopsychosocial conception, highlighting the functioning and disability. When analyzing the ICF qualifiers from interviews 1 and 2 of the SLD group, it can be observed that after speech-language follow-up there was a positive change in the categories of Body Functions, Activities and Participation and Environmental Factors. The ICF allowed the characterization of the functioning and disability in the initial interview^(14)^ and the characterization of how the magnitude of disability decreased with therapy. Participants in the SLD group reported perceiving improvement in their speech and language disorders, and in talking and interacting with different communicative partners. Attitudes of family members, friends and acquaintances, who were considered to have a higher level of barrier in interview 1, became facilitative in interview 2. Thus, the present study indicates that listening to children and adolescents with speech and language disorders about how these factors affect their lives can bring relevant information to speech follow-up.

In Body Functions, participants reported improvement in their articulation functions (d320) and fluency (d330), and reported that family members and friends also said that their speech was easier to comprehend. Results consistent with those of the present study were reported by Thomas-Stonell *et al. ^(^*^12)^, in which parents and clinicians noticed positive changes in Body Functions following speech-language follow-up, confirming its positive effects. In the present study, categories related to intellectual (b117), hearing (b230) and mental functions of language (b167) were not classified as an impairment in interview 1 and did not present changes in interview 2. Although they do not present changes, they are important categories for the characterization of the cases and definition of the therapeutic conduct. ICF allows the description of the functioning and positive aspects of health, and it is important the professionals register categories with no disability too.

Categories of Body Functions are generally considered by SLP in every clinical process, evaluation, therapeutic planning and follow-up reports^(12)^. The impact of speech changes can cause on people’s lives is not always considered by SLP in the same way as observable aspects of fluency or articulation^(12)^. However, if we restrict ourselves to body categories, important aspects related to the social impact of speech and language disorders may not be considered as the focus of follow-up^(17)^.

The professionals who only use Body Function aspects in the therapy would still be limited to a biomedical view, and would not be working with these patients from a biopsychosocial perspective. To evaluate and to register Body Functions should be present in SLP follow-up, but the professional should not be restricted to the components of the body. One of the ways to extend this look beyond the observable aspects of speech and language is to use other components of ICF, like Activities and Participation^(1)^.

In the Activities and Participation component, speaking (d330), conversing (d350), playing (d880), carrying out the daily routine (d230) and relating with others (d710, d730, d750, d760) have become easier for the SLD group. Handling stress (d240) was another category in which participants reported less difficulty. However, for participants with moderate and severe difficulty in articulation and fluency functions, this was still a problem to be considered. Handling stress in particular was a very important item to be characterized by the participants themselves, as only they can tell how they deal with the impact that speech and language disorders have in their lives.

Some of the categories where participants had difficulty in interview 1 and presented positive changes in interview 2 were similar to those reported in other researches: speaking and conversing^(10,14,18)^; interpersonal interactions and relationships^(10,14,18)^ and handling stress^(10)^. However, in one of these studies seven items were reported as having worsened after 7-10 hours of speech-language follow-up^(18)^. It was reported that the children were more reluctant to speak and felt frustrated when they communicated. This result is different from what was found in the present study, that participants reported easier to speak and to converse, and a possible explanation is the difference in speech-language follow-up time. Perhaps the longer follow-up of this research is one of the reasons why no worsening has occurred.

The preference of the TD group for interacting with friends and of the SLD group for relating to family members can be explained by differences in the way both groups interact^(11)^. The interaction of preschoolers with language disorders and without language disorders was studied in the classroom, and differences in relationships and conversation were found among groups. While children without language disorders conversed more with each other, children with language disorders conversed more with adults^(11)^. It is thus suggested that interventions with children should be carried out in schools, promoting a better interaction between them.

Environmental Factors was another component in which changes were observed between interviews 1 and 2. The SLD participants reported fewer barrier attitudes of immediate family members (e410), friends (e420) and acquaintances (e425). One of the factors which may have influenced the attitudes of immediate family members was the group of parents held fortnightly in cases of stuttering. Groups as a therapeutic resource have demonstrated many advantages, however they are still rarely used and there is a shortage of publications in the field^(27)^.

Among the studies that investigated the changes resulting from speech-language follow-up, most addressed the Body Functions and Activities and Participation components^(12,18)^, and few explored Environmental Factors. The results of this study showed that in interview 1, barrier attitudes influenced the choice of people with whom the participants liked to converse, in the daily routine and in handling stress. These data provide important subsidies for a comprehensive, humanized and person-centered speech-language intervention in a biopsychosocial perspective.

One study that addressed the importance of considering Environmental Factors in an outpatient service with children and adolescents in the speech-language pathology evaluation process reported that the most prevalent facilitators were Services, Systems, and Policies; Support and Relationships; and Products and Technology, whereas the barriers were in the categories of Attitudes; Products and Technology. The variable “Change in speech” had a significant association with the categories of chapter 3 - Support and Relationships, and 4 - Attitudes^(21)^.

Getting to know the perception of children and adolescents about their speech and language disorders and the impact they have on daily activities revealed aspects that should be used in the initial characterization of the participant and in the assessment of therapy results^(14)^. Aspects like main communicative partners, difficulties of daily routine, and the attitudes of people, could be better understood because the participants had been heard, and the interview allowed a qualitative deepening of these issues.

Despite the importance of listening to these children and adolescents, most researchers have studied the evaluation of results of therapy from the perspective of therapists and parents ^(12,14,18)^, and used tools with closed questions^(12,15,18)^. Considering that getting to know the perception of affected people can add important information for language therapy, the use of this feature is recommended, and if possible, with questions that allow people to speak about their perceptions from the structure of the ICF.

One limitation of this study is related to the difficult in collecting subjective data and transforming it into a qualifier. Due to the limitation of the explanation about the severity of the difficulty/ impairment/barrier/facilitator, it is difficult to apply a rule to be followed. Despite these limitations, the findings are useful and potentially applicable in the academic and clinical setting, and necessary for widening our view of these children and adolescents.

We highlight as one of the strengths of this article the originality of using the ICF in the speech-language treatment in Brazil. The ICF is still little known and is not widespread in the field, but it has a great potential due to its biopsychosocial approach. Knowledge about children’s and adolescents’ perception on changes in speech-language follow-up is also relevant. After all, few are the studies investigating the ramifications of speech-language therapy from the perspective of the individual with speech and language disorders.

## CONCLUSION

The use of the ICF in this research allowed the professional to broaden perspectives and include categories of the components Body Functions, Activities and Participation and Environmental Factors that go beyond the observable aspects of speech, and it addressed positive changes that speech-language follow-up may bring about: articulation functions, speaking, conversation, social relationships, daily activities, attitudes of people, way of handling stress generated by situations where people act with barrier attitudes that hinder communication. There was a statically significant difference in most of the categories analyzed in both moments.

Analyzing the perception of children and adolescents with speech and language disorders on how they see their own speech and difficulties can provide useful data for speech-language follow-up. Knowing this perception provides information that family members and other professionals cannot always manage to evaluate or perceive in the clinical evaluation which privileges Body components.

The speech-language follow-up that addresses biopsychosocial aspects is one of the factors that reduces the degree of disability of Body Functions, Activities and Participation and Environmental Factors in the perception of children and adolescents with speech and language disorders. Although the short time of follow-up, six months, many changes were reported and registered with the ICF. The ICF is effective throughout the process of speech-language follow-up, and thus it can be used as a parameter to assist the professional in making therapy decisions.

## Footnotes

1 This research initially used the ICF-CY (ICF version for children and young people), in force at the time. In Brazil in 2015^(13)^, there was a merger between ICF and ICF-CY, and most of the categories and codes were kept.
